# The Recognition Algorithm of Two-Phase Flow Patterns Based on GoogLeNet+5 Coord Attention

**DOI:** 10.3390/mi14020462

**Published:** 2023-02-16

**Authors:** Jinsong Zhang, Xinpeng Wei, Zhiliang Wang

**Affiliations:** 1 School of Mechatronic Engineering and Automation, Shanghai University, Shanghai 200444, China; 2 School of Mechanics and Engineering Science, Shanghai University, Shanghai 200444, China

**Keywords:** deep learning algorithm, two-phase flow image, pattern recognition, attention mechanism

## Abstract

The two-phase flow in a microchannel consists of liquid–liquid and gas–liquid material components. The automatic recognition of flow patterns using deep learning approaches has been emerging. This study aimed to improve the recognition accuracy of flow patterns in the two-phase flow images. The different convolutional kernels in the GoogLeNet algorithm extracted the image features with different scales. In order to strengthen the important channel and spatial features, this paper proposes the combined five-layer Coord attention and GoogLeNet algorithm to enhance the accuracy of the new algorithm. The optimized algorithm model was derived from image datasets with different liquid–liquid two-phase flows (NaAlg–Oil, GaInSn–Water), and its accuracy was 95.09% in training and 98.12% in testing. This new model was also applied to predict the flow patterns, with a recognition accuracy of more than 97% in both the liquid–liquid and gas–liquid two-phase flows (water–soybean oil, water–lubricating oil, and argon–water).

## 1. Introduction

A flow system composed of mutually immiscible two-phase substances (with at least one phase being a fluid) is called a two-phase flow. In two-phase flow experiments, the continuous phase and the dispersed phase enter the same channel from different channels, presenting different flow patterns. At present, flow patterns play an important role in the fields of biomedicine, material synthesis, and aerospace [1,2,3]. In the study of two-phase flow images, there are many types of flow pattern; for instance, slug, dripping, and jetting are the flow patterns used to generate monodispersed droplets. The traditional recognition of flow patterns mainly relied on visual observation. Direct visual recognition is effective in low-speed videos, but it is not suitable for high-speed videos. The structure of the two-phase interface is complex, and the flow patterns may be converted in the video, causing some flow patterns to be misclassified. Considering the large number and the inconsistent quality of the flow pattern images, as well as the subjectivity of human observation, scholars have tried to use deep learning to identify flow patterns.

Convolutional neural networks (CNN)—one of the mainstream approaches in deep learning—can recognize and classify the flow patterns in research on two-phase flows. Many scholars have improved the feature extraction ability to obtain better network performance in CNN by increasing the network depth [4,5], enhancing the architecture of the convolution module [6,7,8], and adding new functional units [9]. The mobile network [10,11] and the shuffle network [12,13] increase the deep separable convolution to obtain algorithm models with lower computational cost and higher accuracy. The GoogLeNet algorithm [14] uses convolutional kernels of different sizes to extract the image features at different scales, enhancing the recognition and classification accuracy of the network model.

Adding an attention mechanism to a CNN can improve the recognition accuracy in the image identification. SENet [15] strengthens the important channel features and improves the recognition accuracy. CBAM [16], involving the spatial attention module in SENet, has better recognition and classification results. Coord [17] attention embeds the location information into the channel attention, enhancing the accuracy. LKA [18] embeds the self-attention mechanism into the large kernel convolution for extracting global information.

The research on the recognition algorithms of two-phase flow patterns has mainly focused on the architecture of network algorithms, the optimization of datasets, and the extension of algorithm models. VGG [19,20], ResNet [21,22], and GoogLeNet [23] are the most commonly used algorithms in flow pattern recognition due to their good performance. Some researchers have tried to establish algorithm models to recognize flow patterns using image datasets from different material components [24,25,26], synthetic algorithms [27], and data enhancement algorithms [28]. Nie, F. predicted the flow pattern of nitrogen–liquid nitrogen using a trained algorithm model that was extracted from the tetrafluoromethane–methane flow [23].

To further advance research in this field, this paper introduces an attention mechanism into the GoogLeNet algorithm, improving the recognition accuracy of flow patterns. The optimized model can predict the flow patterns of both liquid–liquid and gas–liquid two-phase flows.

## 2. Experiment and Image Dataset

Our two-phase flow experiments had two kinds of material components (NaAlg–oil, GaInSn–water), with two kinds of microchannels (convergent coaxial and vertical coaxial). The experimental parameters and images are listed in Table 1 and Table 2.

There were four flow patterns (slug, dripping, jetting, and others) in all experiments. The dispersed phases in the slug, dripping, and jetting flow patterns were in the shape of monodisperse droplets. The droplet length of the jetting pattern was obviously smaller than the microchannel width. The droplet length of the dripping pattern was 1.5 times smaller than the inner diameter of the microchannel. Meanwhile, the droplet length of the slug pattern was 1.5 times larger than the inner diameter of the microchannel. In addition to these three flow patterns, the other flow patterns in the two-phase flow were denoted as others.

A total of 24,860 images were collected from the raw videos of the experiments, captured by a high-speed camera. The collection rules for the image datasets were as follows: (1) 15 images with continuous frames were collected from each experiment video, (2) the size of each image was adjusted to be 224 × 224, and (3) similar features were included in each image, such as the channel architecture, the two-phase flows, etc.

The images in this paper had inconsistent sizes; however, the input image pixel in the deep learning algorithm was normalized to be 224 × 224. To match the requirements of the image pixel, the image size was transformed twice from the original pixels to 224 × 224 (Table 3).

In total, the slug pattern occupied 4.2% of the original image datasets. It was obvious that the number of images with a slug pattern was too small to affect the algorithm’s accuracy compared to the other flow patterns. Thus, the data enhancement algorithm was introduced to increase the percentage of slug patterns to 16.9%. As a result, the percentages of the other three flow patterns (dripping, jetting, and others) were 46.66%, 18.97%, and 17.47%, respectively.

## 3. GoogLeNet Algorithm

In general, three-dimensional matrices describe the total image information in deep learning algorithms. The most popular algorithms adopt a matrix of 224 × 224 × 3 as the input parameter, in which 224 × 224 is the size of the image and 3 is the number of red, green, and blue channels. In the processer, many convolution and pooling layers reduce the size of channels (feature maps) and increase the number of channels simultaneously. As a result, the one-dimensional matrix is transformed from the three-dimensional matrices and is set to the softmax classifier, calculating the multi-classification output.

This paper studied the efficacy of four deep learning algorithms in the pattern recognition of two-phase flows.

**Figure 1:**
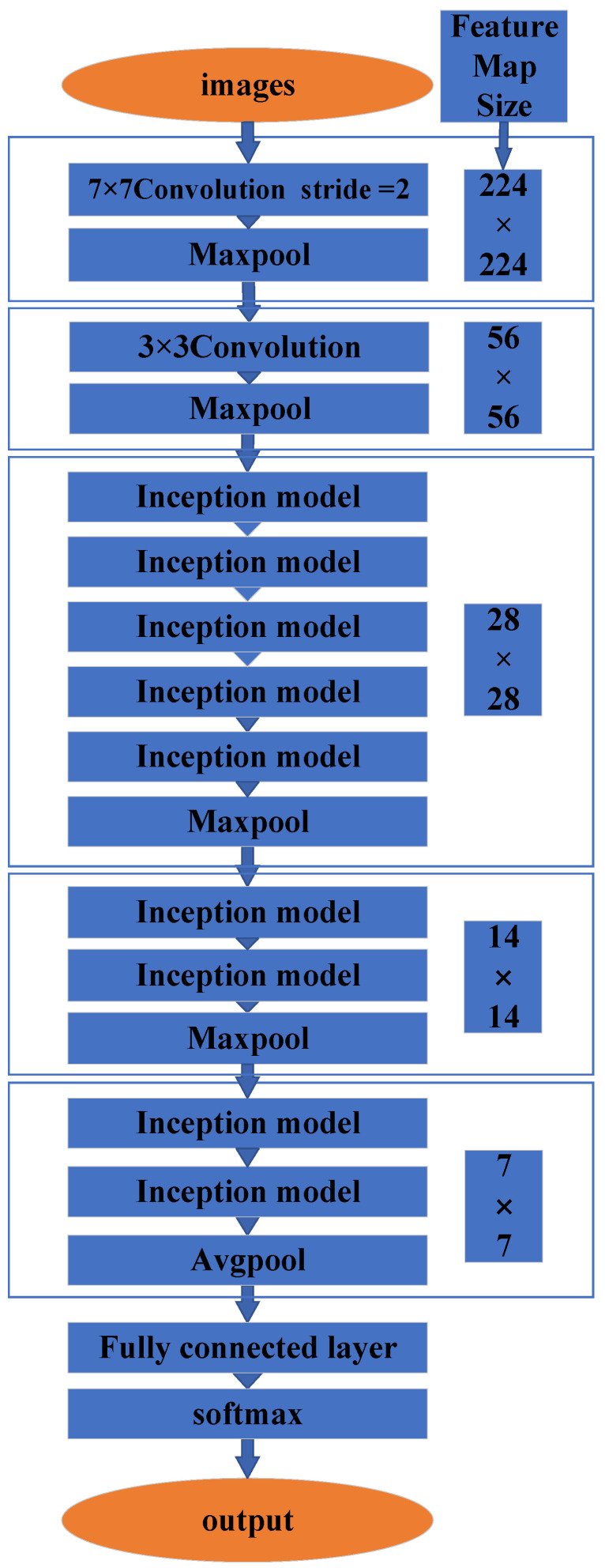
GoogLeNet architecture.

GoogLeNet [14]: The GoogLeNet algorithm (Figure 1) is 22 layers in depth for parameters or 27 layers in depth for pooling counting. Its first four layers are the convolutional layers and the pooling layers. Beneath the algorithm layer, the GoogLeNet algorithm creates the inception module (Figure 1). For example, there are two layers of the inception module in the feature maps of 28 × 28 and 7 × 7, and five layers of the inception module in the feature map of 14 × 14.

**Figure 2:**
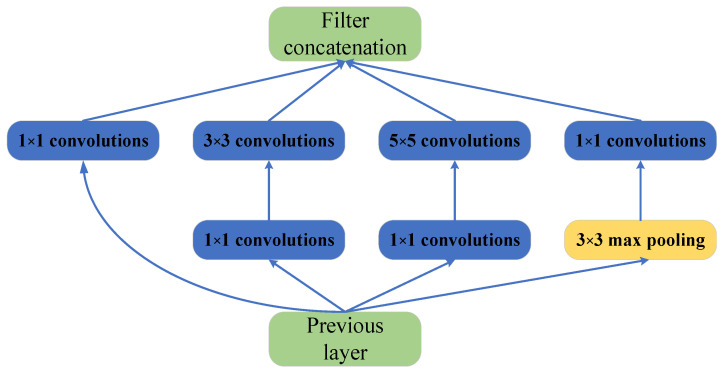
Inception architecture.

The inception module connects the multiple convolution kernels and the maximum pooling in parallel. It consists of three convolutions with different sizes and a maximum pooling layer (Figure 2). The calculation of the multiple convolution kernels increases explosively as the number of algorithm layers increases. The inception module introduces the 1 × 1 convolution to reduce the computational cost. The parallel 1 × 1, 3 × 3, and 5 × 5 convolutions can extract richer features in a layer, resulting in higher recognition accuracy.

VGG 16 [5]: The Visual Geometry Group Network (VGG16) has a total of 13 convolutional layers and 3 fully connected layers. All of the convolution and pooling layers adopt 3 × 3 convolutional kernels and 2 × 2 pooling kernels.

ViT [29]: Vision Transformer (ViT) introduces the Transformer module. The input of the Transformer module is a one-dimensional matrix transformed from three-dimensional matrices in the algorithm. The whole image being flattening into a one-dimensional matrix addresses the issue of huge computational cost. ViT divides the image into 16 windows and flattens it into a one-dimensional matrix to reduce the amount of calculation.

Swin-T [30]: Swin-Transformer (Swin-T) also applies the Transformer module. Unlike ViT, which calculates the self-attention in the image, Swin-T calculates the Transformer in each small window first, and then merges the small windows into large windows. After the three iterations of window merging and calculation, the output of the Transformer is sent into the fully connected layer and the softmax layer.

**Figure 3:**
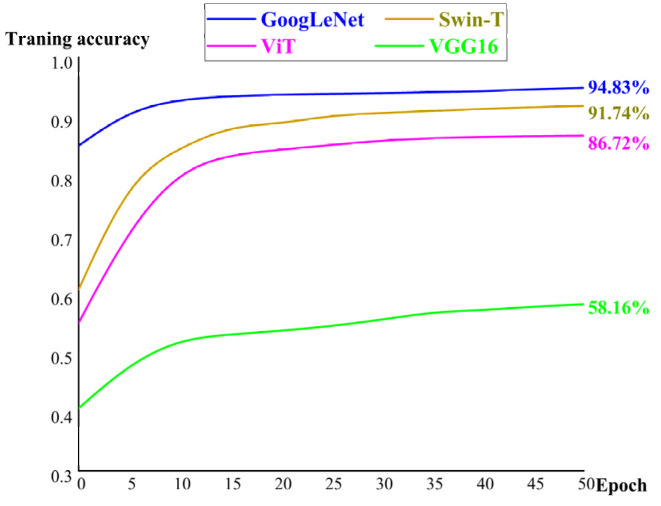
Training accuracy of four algorithms (VGG, ViT, Swin-T, and GoogLeNet).

For comparison, VGG16, GoogLeNet, ViT, and Swin-T were trained to recognize the flow patterns after 50 iterations based on our image datasets in the liquid–liquid two-phase flows (Figure 3). The green solid line represents VGG16, with a training accuracy rate of 58.16%. The yellow solid line represents ViT, with a training accuracy rate of 86.71%. The blue solid line represents Swin-T, with a training accuracy rate of 91.74%. The red solid line represents GoogLeNet, with a training accuracy rate of 94.83%. In all experimental results, GoogLeNet showed the best recognition accuracy for the two-phase flow patterns.

## 4. Coordinate Attention

In order to improve the recognition accuracy of GoogLeNet beyond 94.83%, this paper implanted an attention mechanism into the GoogLeNet algorithm.

The principle of an attention mechanism is to locate the interesting information and suppress the useless information by changing the weights of different areas. Attention has been widely used in computer vision and natural language processing.

**Figure 4:**
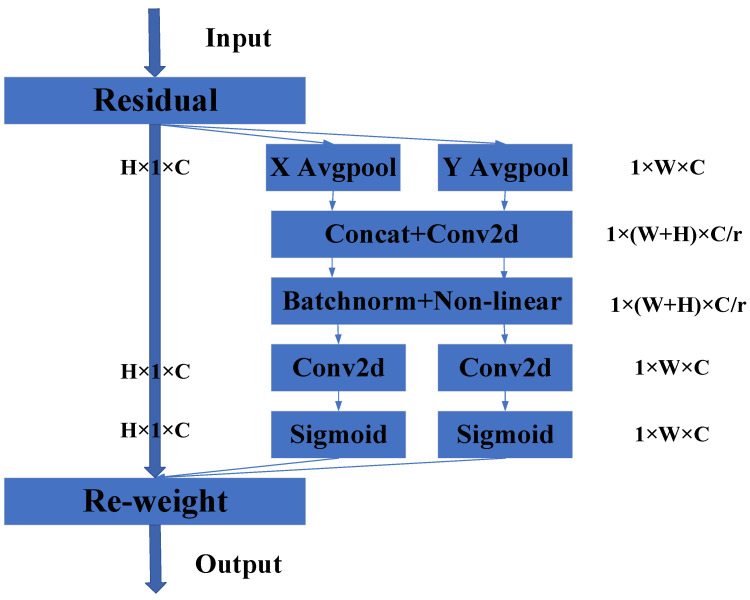
Coordinate attention architecture.

The following four common attention were discussed: 

Coord: Coordinate attention (Coord) embeds the positional information into the channel attention, and strengthens the channel and spatial features (Figure 4).

As shown above, Coord attention implements two pooling kernels, (*H*, 1) or (1, *W*), to encode spatial information in the horizontal channel and the vertical channel, respectively. Equations (1) and (2) give the calculation equations:

(1) $$Z_{c}^{h}(h)=\frac{1}{W}\sum_{0\leq i<W}x_{c}(h,i)$$

(2) $$Z_{c}^{W}(w)=\frac{1}{H}\sum_{0\leq i<H}x_{c}(j,w)$$

where *x_c_* is the input of the *c*-th dimension, *Z(h)* is the *H*-dimensional output of the *c*-th channel, and *Z*(*w*) is the *W*-dimensional output of the *c*-th channel.

The contact layer and 1 × 1 convolution compress the channel in the spatial dimension and encode the spatial information in the vertical and horizontal directions through the batch-norm. The output is transformed into a pair of feature maps by the convolution transformation function. The convolution transformation function is

(3) $$F=\delta (F_{1}(Z^{h},Z^{w}))$$

where *F* is *R^C^*^/r*(*H* + *W*)^, and *δ* is the nonlinear activation function.

Finally, the spatial features in the horizontal and vertical channels are calculated separately through 1 × 1 convolution, and then the features of the convolutions are put together to recalculate the weights. The formulae of output Y are

(4) $$G^{h}=\sigma (F_{h}(f_{h}))$$

(5) $$G^{w}=\sigma (F_{w}(f_{w}))$$

(6) $$y_{c}(i,j)=x_{c}(i,j)\times G_{c}^{h}(i)\times G_{c}^{w}(j)$$

where *f_h_* is *R^C^*^/r*H^, and *f_w_* is *R^C^*^/r*W^.

Coord attention accounts for both the channel-to-channel relationships and the location information. It captures not only the information across channels, but also the direction-aware and position-sensitive information, which can accurately locate and identify the target areas.

CBAM: The convolutional block attention module (CBAM) is divided into channel and spatial modules. The channel attention module compresses the feature map in the spatial dimension. The spatial attention module compresses the channel in the spatial dimension. CBAM sequentially generates an attention map in two independent dimensions (channel and space).

LKA: Large kernel attention (LKA) introduces self-attention into the large convolution kernels. The convolution of large kernel size is decomposed into the depthwise convolution, the depthwise empty convolution, and the channel convolution. LKA establishes the correlation of each point, generating the attention map in the large kernel convolution, which realizes the adaptability of the channel dimension and the spatial dimension.

SENet: Squeeze-and-Excitation Networks (SENet) are divided into squeeze and excitation modules. The squeeze module performs the feature compression in the spatial dimension, turning the 2D feature channel into a real number. The excitation module calculates the weights and correlations between each feature channel.

## 5. Optimized Algorithm Results and Discussion

### 5.1. Optimized Algorithm Architecture

**Figure 5:**
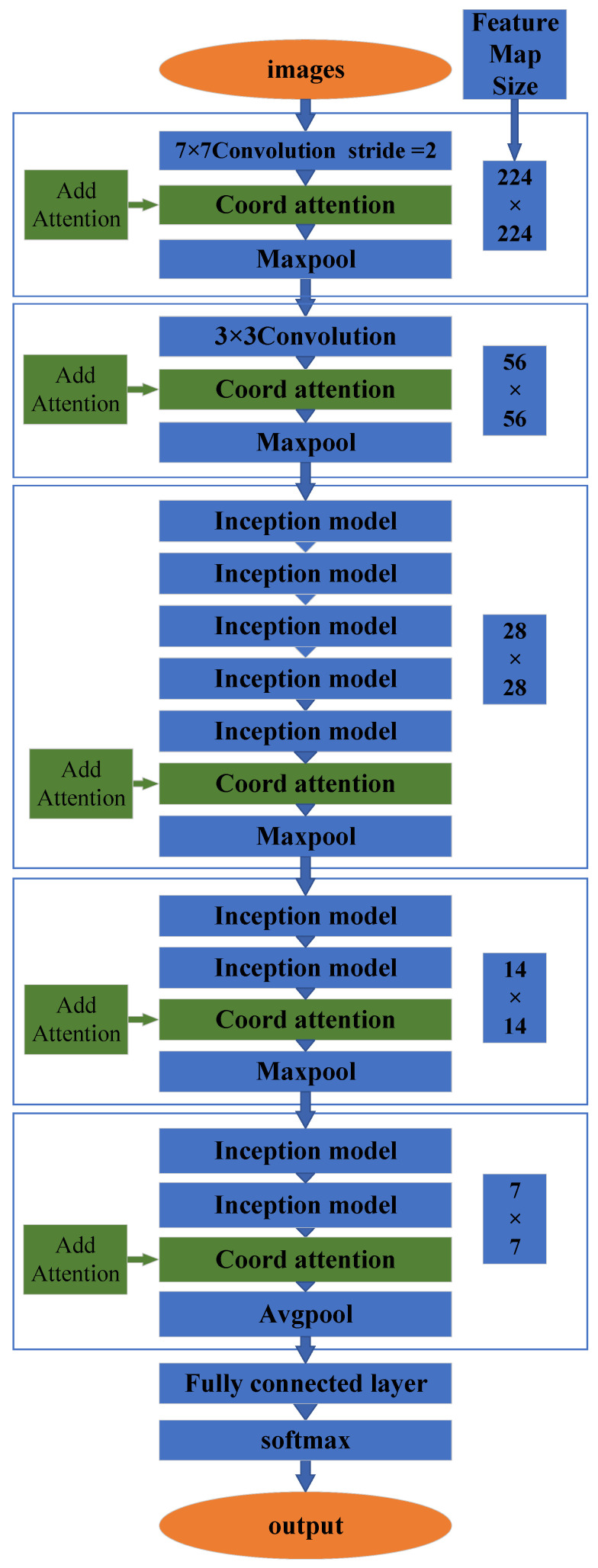
Architecture of GoogLeNet+5 Coord attention.

Four kinds of attention (Coord, CBAM, LKA, and SENet) were introduced into the GoogLeNet algorithm for the training and validation datasets. The innovative architectures possessed five layers of attention to be embedded in each map, with sizes of 224 × 224, 56 × 56, 28 × 28, 14 × 14, and 7 × 7. Figure 5 illustrates one architecture of the four attention, and Table 4 gives the results of their recognition accuracy. Compared with the three other attention, the accuracy of Coord attention was the best. This new GoogLeNet+5 Coord attention algorithm was proposed to recognize flow patterns.

GoogLeNet focuses on the local features extracted by the convolution kernels with different sizes; however, GoogLeNet is not good at global feature extraction. Coord attention can extract global features and also strengthen the important spatial and channel features. Coord attention compensates for the lack of global, spatial and channel features in GoogLeNet due to its computational reduction. Introducing Coord attention into the GoogLeNet algorithm improved the performance in recognizing the flow patterns, with a high accuracy and low loss in the training and validation steps.

In our two-phase flow images, the GoogLeNet algorithm easily extracted the local features, especially for the droplet features. Additionally, Coord attention was supplemented to extract the global features of images, such as the background and microchannel features. The innovative GoogLeNet+5 layers of the attention algorithm converged the local features of two-phase flows and the global features of the background and microchannels, which distinguished the background features clearly to recognize the flow patterns with a higher accuracy.

### 5.2. Training and Testing Results

**Figure 6:**
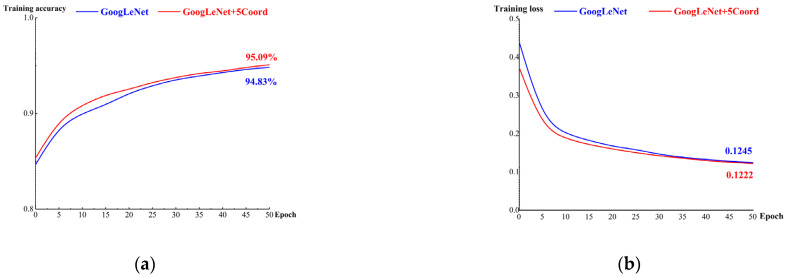
Accuracy and loss of the training dataset when applying two algorithms: (**a**) training accuracy; (**b**) training loss.

The dataset was separated into model and test datasets at a ratio of 8:2, and the model dataset was also separated into training and validation datasets at a ratio of 8:2. For the training dataset, setting the batch size to 32, the learning rate to 0.0001, and the iteration number to 50, the recognition results of GoogLeNet and GoogLeNet+5 Coord are plotted together in Figure 6.

The blue solid curve represents the training results of GoogLeNet, with an accuracy of 94.83% and loss of 0.1245. The red solid curve represents the training results of GoogLeNet+5 Coord, with an accuracy of 95.09% and loss of 0.1222. This indicates that the new algorithm, GoogLeNet+5 Coord, had a higher training accuracy and lower training loss than the traditional GoogLeNet algorithm—by 0.26% and −0.0023, respectively.

**Figure 7:**
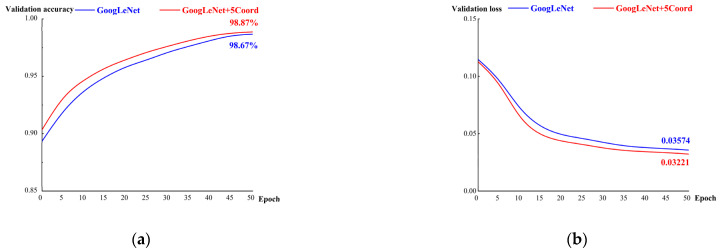
Accuracy and loss of the validation dataset when applying two algorithms: (**a**) validation accuracy; (**b**) validation loss.

Figure 7 presents the validation results of GoogLeNet+5 Coord and GoogLeNet. The blue solid curve represents the validation result of GoogLeNet, with an accuracy of 98.67% and loss of 0.03574. The green solid curve represents the validation result of GoogLeNet+5 Coord, with an accuracy of 98.87% and loss of 0.03221. The new GoogLeNet+5 Coord had better validation results for accuracy (+0.2%) and loss (−0.00353) than the traditional GoogLeNet algorithm.

**Figure 8:**
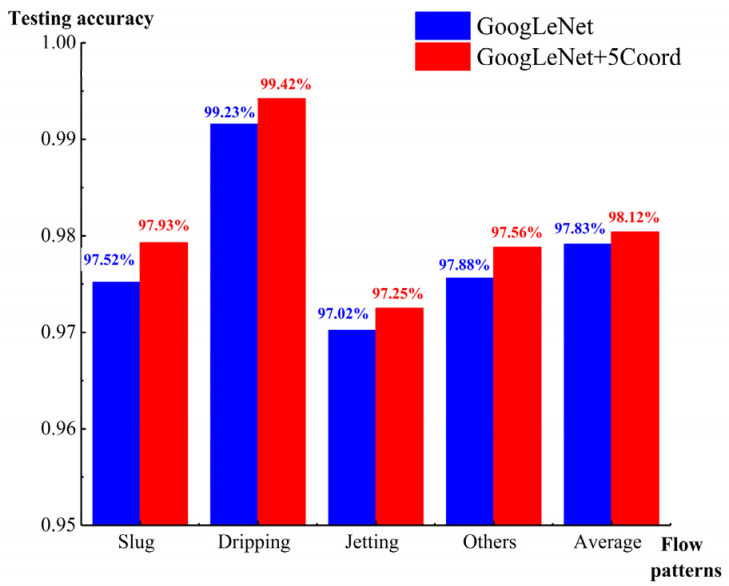
Testing accuracy of GoogLeNet+5 Coord and GoogLeNet.

After the establishment of the algorithm model, the remaining 20% of the data were used in the testing dataset to test the model precision of GoogLeNet+5 Coord and GoogLeNet (Figure 8). For 50 iterations with the testing dataset, the average recognition accuracy of all images when applying GoogLeNet+5 Coord was 98.12%, which was about 0.29% higher than that of GoogLeNet.

### 5.3. Prediction Results

The optimized algorithm model with a higher accuracy (>98%) was derived from the image datasets with liquid–liquid two-phase flows (NaAlg–oil, GaInSn–water).

This paper extended the model of GoogLeNet+5 Coord to predict the flow patterns in the different gas–liquid and liquid–liquid two-phase components. Similarly, the datasets contained 600 images with the four flow patterns (Table 5). The average accuracy was 97.65% in the prediction of gas–liquid flow patterns.

**Figure 9:**
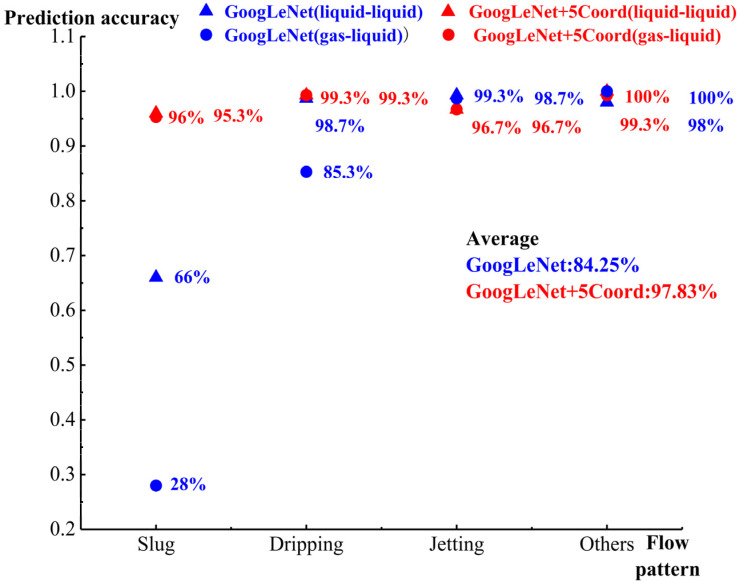
Prediction accuracy of two-phase flows.

Figure 9 depicts the prediction accuracy of the two algorithms in both the liquid–liquid and gas–liquid two-phase flows. GoogLeNet+5 Coord had better performance than GoogLeNet. The new algorithm model could accurately identify the four flow patterns, with an average accuracy of 97.83%. GoogLeNet had poor accuracy in identifying the slug flow pattern, with an average accuracy of 84.25%. This shows that GoogLeNet+5 Coord had higher accuracy and better consistency than GoogLeNet.

**Figure 10:**
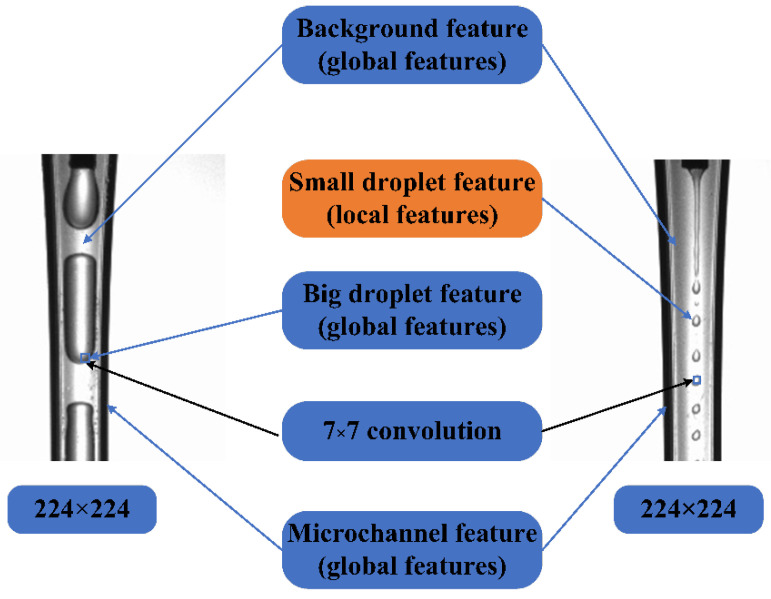
Local features and global features of slug and jetting images.

In our two-phase flow images, the GoogLeNet algorithm easily extracted the local features, especially for the small droplet features. For example, for jetting, the largest convolution of GoogLeNet was 7 × 7, which was approximate to the size of a droplet. Therefore, the prediction accuracy for jetting in GoogLeNet was higher than in GoogLeNet+5 Coord. Moreover, the convolution of 7 × 7 was much smaller than the droplet size of the slug flow pattern, and the recognition accuracy of the slug pattern in prediction was poor. Coord attention was supplemented to extract the global features of images, such as the background features, large droplet features, microchannel features, etc. The innovative GoogLeNet + 5 layers of attention algorithm converged the local features of the two-phase flows and the global features of the large droplets, the background, and the microchannels to accurately predict the four flow patterns (Figure 10).

## 6. Conclusions

This paper researched flow pattern recognition from image datasets of gas–liquid and liquid–liquid two-phase flows.

1. Compared with the VGG16, ViT, and Swin-T algorithms, the GoogLeNet algorithm had a higher accuracy in recognizing and classifying flow patterns.

2. Different attention were introduced to improve the recognition accuracy, and it was found that the optimal algorithm was GoogLeNet+5 Coord, which strengthened the important channel and spatial features and extracted the two-phase flow features simultaneously.

3. The optimized GoogLeNet+5 Coord algorithm was trained from the data of different liquid–liquid two-phase flows, and it could predict the liquid–liquid and gas–liquid two-phase flow patterns with a high accuracy of more than 97%.

4. The optimized algorithm model was a normalized model for flow pattern recognition in both liquid–liquid and gas–liquid two-phase flows.

## Figures and Tables

**Table 1:** Experiment parameters of two-phase flows.

Material Components	Microchannel Type	Convergence Angle	Dispersed Phase	Continuous Phase	Experimental Groups
NaAlg –Oil	Convergent coaxial	9°	NaAlg (10~480 mL/h)	Soybean oil (1~10 mL/h)	773
Oil– NaAlg	Convergent coaxial	9°	Soybean oil (1~250 mL/h)	NaAlg (1~10 mL/h)	734
NaAlg –Oil	Vertical coaxial	--	NaAlg (10~480 mL/h)	Soybean oil (1~10 mL/h)	658
Oil– NaAlg	Vertical coaxial	--	Soybean oil (1~250 mL/h)	NaAlg (1~10 mL/h)	439
GaInSn–water	Vertical coaxial	--	GaInSn (7~108 mL/h)	Water (36~900 mL/h)	132

**Table 2:** The definition of flow patterns with different images.

Flow Pattern	Features	Experiment Images			Quantity of Images
Slug	Monodisperse droplets with a bullet-like or plunger-like shape, and the droplet length is 1.5 times larger than the inner diameter of the microchannel	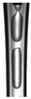	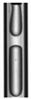	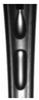 ^1^	3624
Dripping	Monodisperse droplets with an ellipsoid or spherical shape, and the droplet length is 1.5 times smaller than the inner diameter of the microchannel	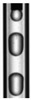	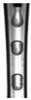	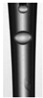 ^1^	10,385
Jetting	Monodisperse droplets with a nearly spherical shape, and the droplet length is significantly smaller than the inner diameter of the microchannel, with fast generation frequency and a stretching neck	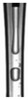	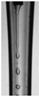 ^1^	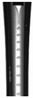 ^1^	4366
Others	Other flow patterns	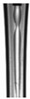	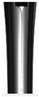 ^1^	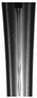 ^1^	4717

^1^ Represented blurred image.

**Table 3:** Original pixels transformed into uniform pixels.

	Slug	Dripping	Jetting	Others
Images of original pixels	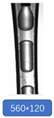	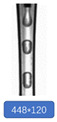	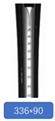	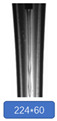
Images of uniform pixels	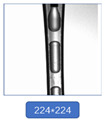	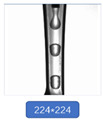	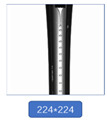	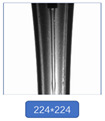

**Table 4:** The accuracy of the GoogLeNet+5 layers with different attention.

Algorithm	Training Accuracy (%)	Validation Accuracy (%)
GoogLeNet	94.83	98.67
GoogLeNet+5 Coord	95.09	98.87
GoogLeNet+5 CBAM	94.97	98.72
GoogLeNet+5 LKA	94.71	98.54
GoogLeNet+5 SENet	95.04	98.67

**Table 5:** Experiments for flow pattern prediction.

Material Components	Dispersed Phase	Continuous Phase	Experimental Groups	Images
Oil–water	Vegetable oil	Water	59	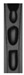 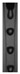 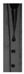 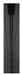
Oil–water	Lubricating oil	Deionized water	52	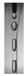 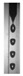 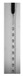
Argon–water	Argon	Water	93	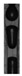 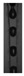 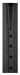 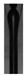
